# Major challenges and barriers for successful management of health exercises in emergencies and disasters: findings from a qualitative conventional content analysis study

**DOI:** 10.1186/s12873-025-01360-3

**Published:** 2025-10-14

**Authors:** Hojjat Farahmandnia, Majid Sartipi, Ali Nasiri, Ali khosravizad, Asiye Aminafshar

**Affiliations:** 1 Health in Disasters and Emergencies Research Center, Institute for Futures Studies in Health, Kerman University of Medical Sciences, Kerman, Iran; 2 Infectious Diseases and Tropical Medicine Research Center, Research Institute of Cellular and Molecular Sciences in Infectious Diseases, Zahedan University of Medical Sciences, Zahedan, Iran; 3 Department of Biostatistics and Epidemiology, School of Health, Zahedan University of Medical Sciences, Zahedan, Iran; 4 Health Management Research Center, Baqiyatallah University of Medical Sciences, Tehran, Iran; 5 Communicable Diseases Research Center, Ilam University of Medical Sciences, Ilam, Iran; 6 Health Services Management Research Center, Institute for Futures Studies in Health, Kerman University of Medical Sciences, kerman, Iran; 7 Student Research Committee, Kerman University of Medical Sciences, Kerman, Iran

**Keywords:** Challenges, Barriers, Health, Exercises, Emergency, Disaster

## Abstract

**Introduction:**

Enhancing health system preparedness requires the design, implementation, and evaluation of disaster exercises. Conducting these exercises improves preparedness planning and promotes staff awareness and capabilities in responding to emergencies and disasters. Given the existing challenges in designing and executing disaster exercises within the health sector, this study aims to identify the main challenges and barriers to the successful management of health sector disaster exercises.

**Method:**

This study was a conventional qualitative content analysis. Data were collected through purposive sampling through in-depth, semi-structured individual interviews with 23 managers from various health sectors. Graneheim and Lundman’s conventional content analysis was employed to analyze the data, and Lincoln and Guba’s recommendations were utilized to ensure the trustworthiness of the data.

**Result:**

A total of 170 challenges (initial codes), organized into one them, five main categories and 15 subcategories, emerged after several rounds of data analysis and summarization, considering both similarities and differences. These main categories included “Organizational management”, “structural and administrative system”, “Ineffective policymaking”, “Cultural sensitivity and social participation”, and “organizing exercise steps”.

**Conclusion:**

Understanding the obstacles and challenges in implementing discussion-based and operations-based exercises in emergencies and disasters derived from the experiences of exercise implementers in the health system can help health planners and managers proactively address these obstacles and by considering these challenges and striving to reduce them before designing an exercise they can maintain and improve preparedness of their community’s healthcare system through the implementation of successful and effective exercises.

## Introduction

Disasters are unexpected events that cause economic and human losses and can disrupt the functions of a society. Given the frequent disasters worldwide, many countries strive to enhance their preparedness for disasters [1]. Healthcare systems must possess a desirable level of resilience to provide an appropriate response to disasters. A prerequisite for achieving favorable resilience in the face of disasters is having preparedness for such incidents [2]. The lack of preparedness in the healthcare system to deal with emergencies and disasters leads to delays in treatment, the collapse of healthcare services, and the loss of specialized functions [3]. During the preparedness phase, it is essential to focus on developing empowerment measures for healthcare workers and conducting performance evaluations to enhance care during emergencies [4].

Training and exercises are among the elements expected by the Federal Emergency Management Agency (FEMA) and the National Emergency Management Association (NEMA) in preparedness programs [5]. One way to enhance and maintain the preparedness of the healthcare system is through the implementation of exercises [6, 7]. In our country, Iran, the National Crisis Management Strategy Document, the National Disaster Risk Reduction Program, the National Preparedness and Response Program, and the National Reconstruction and Rehabilitation Program have been developed at the national level to manage disaster and emergency risks. In the field of health, the National Health System’s Disaster Response Program, the Hospital Disaster Preparedness Program, the Health System’s Disaster Response Framework, the National Exercise Program, the National Risk Assessment Toolkit, and other important programs have also been developed [8–11]. Exercises are simulations of potential and hypothetical incidents designed to evaluate and strengthen operational plans, leading to improved planning and enhanced knowledge and skills among disaster response teams. In essence, exercises provide learning opportunities for health systems to assess how different components of the health system function and integrate when facing incidents and disasters, and to identify and address systemic strengths and weaknesses. Additionally, exercises offer opportunities to enhance coordination and collaboration among various stakeholders [12].

Exercises provide a training opportunity for health systems to assess how different parts of the health system (performance) integrate and function in the face of emergencies and disasters [13]. Exercises help individuals sharpen their mental and physical skills for similar situations, leading to more effective responses in real-life incidents. They also enable the testing and validation of policies, plans, and procedures, as well as the training of staff on their roles and responsibilities, the improvement of individual performance, and enhanced communication and coordination across organizations [14, 15].

Continuous and planned exercises lead to improved interdepartmental coordination, enhanced decision-making, and the identification of system gaps before disasters occur [16]. Considering the importance of implementing exercises to strengthen health system preparedness for disasters, the absence of a systematic approach to planning, designing, and implementing these exercises within organizations can result in the waste of organizational resources and an inadequate response during a disaster [17]. Securing financial resources from managers and believing in the effectiveness of training in improving personnel’s functional knowledge, utilizing plausible and realistic scenarios, anticipating expert expectations for training evaluation, conducting briefing sessions before and after training, and implementing continuous and regular training will improve personnel performance [17].

### Literature review

The study by Emaliyawati et al. demonstrated that exercise strengthens and enhances teamwork, improves coordination and communication among participants, and boosts knowledge, attitude, and self-confidence in health workers [18]. Hampson et al., as well as Porthouse, Alexander et al., demonstrated that preparedness is a core component of incident management, requiring planning, staff training, community education, and exercises and evaluation [19, 20]. Syahirul Alim et al., in a study, examined the effectiveness of conducted training and exercises using three components: (a) administering pre-tests and post-tests to measure the amount of knowledge gained during the training session; (b) evaluating skills through observation during disaster exercises using a performance checklist; and (c) conducting interviews to gather responses and feedback from participants regarding the training and exercises. This study confirmed the effectiveness of the training and exercise program in improving participants’ preparedness level in facing disasters. However, it emphasized the expansion of educational programs and the dedicated evaluation of short-term and long-term training programs to enhance disaster preparedness [21]. Zhuge et al. identified a shortage of human resources, a lack of financial resources and equipment, weak organizational coordination for conducting practical exercises, unequal power distribution, and an organizational hierarchy as barriers to the continuous implementation of exercises [22]. Sheikh Bardsiri et al. also emphasized the necessity of inter-organizational collaboration in their study for developing and testing response plans through standardized exercises to enhance health system preparedness [23]. In another study, Sheikhbardsiri et al. used a qualitative approach to identify six main categories for the effective implementation of exercises in Iran: coordination and information management, standards and indicators, process guidance and control, logistics and procurement management, treatment operations management, and health operations management. These categories should be considered as guidance for conducting health-related exercises [24]. In their study, Shirazi et al. demonstrated that tabletop exercises are both cost-effective and efficient for enhancing disaster preparedness due to their lower resource requirements. These exercises are well-suited for maintaining readiness in dynamic healthcare environments, significantly boosting coordination and trust among different sectors, and improving inter-organizational communications [25].

### Main problem, necessity, and objectives of the study

Conducting health exercises in emergencies and disasters significantly impacts the enhancement of preparedness, inter-sectoral coordination, and strengthening resilience. However, evidence indicates that organizing health exercises across various organizational levels faces numerous challenges. These challenges in conducting health exercises are complex and multi-layered, necessitating the exploration of expert perspectives. Qualitative studies serve as a tool for a deep understanding of intricate phenomena. Therefore, by gathering participants’ experiences, perceptions, and viewpoints, a profound sense of the barriers to exercise can be achieved. Failing to identify and comprehend these challenges will result in an inability to achieve the expected level of preparedness for responding to emergencies and disasters effectively. This research aimed at identifying the main challenges and barriers to the successful management of health sector disaster exercises. This study provides a standardized framework for health managers preparing to design disaster exercises in the health system. One of the crucial innovations of this study is that we identified the barriers and challenges to the successful implementation of exercises tailored to the cultural structure of the Iranian healthcare system. These challenges and barriers may differ from those faced by other countries’ healthcare systems. It recommends that managers proactively address potential challenges and obstacles prior to designing, implementing, and evaluating the required exercises.

## Method

### Study design and setting, and participants

This study employs a qualitative design with a conventional content analysis approach to extract the main challenges and barriers to the successful management of health sector disaster exercises in 2025. Qualitative content analysis is a suitable method for understanding topics where information is limited and dispersed [26, 27]. This study was conducted through interviews with key informants, using purposive sampling with 23 individuals who were selected from 10 provinces of Iran, including Tehran, Shiraz, Isfahan, Mashhad, Tabriz, Yazd, Bushehr, Golestan, Kerman, and Mazandaran that had rich experience in the filde of designing, conducting and evaluation of disaster exercises (discussion-based and operations-based) in emergencies and disasters. These participants included managers from the health system including treatment sectors (prehospital and hospital), logestic sectors, health care sectors, disaster risk reduction office and Emergency Operations Center (EOC).

### Inclusion and exclusion criteria of the study

The inclusion criteria for the study were having rich experience in the filde of designing, conducting and evaluation of disaster exercises in emergencies and disasters and willingness to participate in the study.The exclusion criteria were lack of interest in participating in the research, as well as the inability or lack of sustained cooperation during the research implementation.

### Data collection instruments

In the present study, the data collection method needed to facilitate an in-depth investigation into the experiences, dimensions, and identification of challenges and barriers to the successful management of health sector disaster exercises. Interviews are used to collect information that is not directly observable. This method typically explores individuals’ motivations, feelings, attitudes, actions, and experiences. An interview consists of the interviewer’s oral questions and the research participant’s oral responses [28]. This qualitative study employed in-depth and semi-structured interviews, starting with general questions. The research team members were actively involved in developing this interview guide. Two pilot interviews were conducted with individuals outside the leading sample group to examine the conceptual validity and ensure the clarity and meaning of the questions from the participants’ perspective. The feedback from these interviews, along with the research team’s comments, served as the basis for the final revision of the interview guide. Depending on the progress of the conversation, probing and guiding questions were asked to deepen the participants’ understanding of their experiences and perspectives. Interviews were conducted from April to June 2025 in a location agreed upon by the participants and where they felt comfortable. The main questions include:

- Please describe a discussion-based or operational-based exercise in which you have previously participated, focusing on its beneficial aspects.
- From your perspective, what obstacles and challenges exist for designing a standardized disaster exercise?
- What obstacles and challenges have you faced in implementing a discussion-based and operations-based exercise with global standards?
- What weaknesses and gaps exist in the standardized evaluation of disaster exercises in the health sector?

Exploratory questions, such as “Can you provide an example of this issue or challenge you faced?” or “Could you elaborate further on this topic?” were utilized to gain deeper insights into the challenges and concepts discussed. The approximate duration of the interviews was between 18 and 50 min (average 45.3 min).were utilized to gain deeper insights into the challenges and concepts discussed. In this study data collection continued until saturation was reached. While a consensus on the precise definition of data saturation remains elusive, and a systematic approach to its implementation is lacking, it is generally understood as the iterative process of incorporating new participants into the dataset until data redundancy emerges [29]. Saturation is achieved when the addition of further data yields diminishing returns, failing to contribute novel insights to the existing dataset. The objective of data saturation is to ensure replicability within categories; replication serves to validate findings and ensure thorough comprehension and completeness. In essence, qualitative researchers employ progressive case selection, continuing until data saturation is achieved, without predetermining the total number of participants [30]. In this study, data saturation was determined after the 21th interview. To ensure saturation, two additional interviews were conducted, and no new codes were identified.

### Ethical consideration

Ethical considerations in this study included explaining the importance, objectives, and methods, as well as providing optional participation in the study, recording the interviews, maintaining data confidentiality at all stages, obtaining written consent, and making a mutual decision about the time and place of the interview. Individuals were assured that the obtained information would be used solely for research purposes. Throughout the study, researchers took steps to anonymize data, protect participants’ identities, and ensure that personal information remained secure.

### Data analysis

**Fig. 1:**
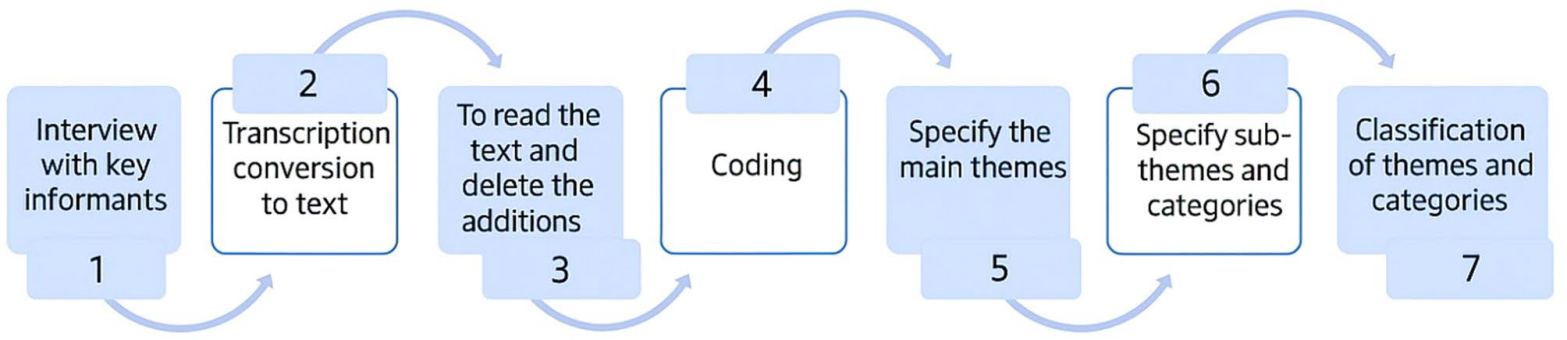
The steps of qualitative data analysis

Data analysis was conducted in five steps, following the Graneheim and Lundman approach [31]. Initially, the recorded interviews were transcribed verbatim, and the transcriptions were meticulously compared with the original audio files to ensure accuracy. In the second step, the entire text was read multiple times to gain a comprehensive overview. In the third step, meaning units were identified, condensed, and coded for each interview. These meaning units consisted of words, sentences, or paragraphs that contained relevant aspects in terms of content and context. In the fourth step, codes with similar meanings were categorized under broader subcategories. Finally, in the last step, by comparing the subcategories with each other and through deep and careful reflection, the latent content within the data was presented as the main categories. The qualitative data analysis was facilitated using MAXQDA software, version 20. The steps of qualitative data analysis are specified in Fig. 1. The figures and charts used in this article were designed using the Napkin AI App.

### Reliability and validity

The criteria for scientific accuracy in qualitative research, as proposed by Guba and Lincoln, were employed to ensure the rigor and trustworthiness of the data. These criteria encompass four stages: credibility (or believability), dependability (and consistency of data), confirmability, and transferability [32]. Credibility was established using methods such as prolonged engagement and persistent observation, maximum variation sampling about participants’ education and workplace across different levels, peer debriefing (review of codes and categories by research colleagues), and member checking (review of codes and categories by study participants). To ensure confirmability, the research findings needed to be verified and validated by the research team to minimize the potential influence of the researcher’s perceptions on the research process. The research team confirmed the research process and its findings in this study.

## Results

### Demographic information of participants

The study participants consisted of 23 who had experience conducting disaster exercises. Among the participants, 19 were male, and four were female. Fourteen participants held bachelor’s degrees, six had master’s degrees, and three held doctoral degrees. Of the 23 managers, six were from the prehospital area, four were from the Emergency Operations Center (EOC), nine were from the hospital area, two were from the logistics department of the University of Medical Sciences, and two were from the Disaster Risk Reduction Office. The demographic characteristics of the participants are presented in Table 1.

**Table 1:** Demographic information of participants

Demographic Characteristics	Subcategory	Number (%)
Gender	Male Female	19 (82.61) 4 (17.39)
Age (Years)	25–35 36–45 ≥ 46	5 (21.74) 12 (52.17) 6 (26.09)
Work Experience (Years)	≤ 10 11–20 21–30	6 (26.09) 11 (47.83) 6 (26.09)
Organizational positions	Prehospital Hospital Logistic EOC Disaster Risk Reduction Office	6 (26.09) 9 (39.13) 2 (8.69) 4 (17.39) 2 (8.70)
Educational Status	Bachelor’s Master’s Doctoral Degrees	14 (60.87) 6 (26.09) 3 (13.04)

**Table 2:** Major challenges and barriers for successful implementation of health exercises in emergencies and disasters

Theme	Main Categories	Sub Categories	Example of codes
Challenges and Barriers for Successful Implementation of Health Exercises in Emergencies and Disasters	Organizational management	Managerial competence and expertise deficits	-Putting untrained people in charge -Insufficient professional training -No background or certifications in disaster management -A lack of innovative thinking
		Insufficient strategic planning and foresight	-Lack of managerial motivation - Overemphasis on response rather than prevention - Failure to evaluate exercises or Insufficient evaluation of exercises
		Frequent management turnover and organizational instability	-Frequent turnover of personnel or high leadership turnover -Lack of knowledge transfer to new managers -Missed opportunities from letting go of experienced managers -Erosion of stability and continuity in training programs
	Structural and administrative system	Financial constraints	-insufficient funding -Administrative complexities in obtaining permits -Wastage of resources
		Inadequate inter-agency coordination	-Organizational and inter-organizational breakdown - Lack of coordination with partner organizations
		Complex bureaucratic processes	-Bureaucratic obstacles to drill implementation -Lengthy and complex administrative procedures for approvals and funding -Negative impact of bureaucracy on motivation and outcomes -Excessive paperwork -Lack of unified procedures and poor intra-organizational coordination
	Ineffective policymaking	Political influence in the appointment of emergencies and disasters managers	-Political appointments of unqualified individuals.
		Predominant reactive disaster response approach instead of preparedness	-Limited understanding of disaster management - Resources allocated to the response phase instead of preparedness
		Leadership’s undervaluation of preparedness exercises	-Viewing drills as a performative exercise -Disregarding the true objectives of the drill - Belief among managers and staff that exercises are an unnecessary activity
	Cultural sensitivity and social participation	Limited community engagement	-Excluding public participation
		Conflicts with established local customs and practices	-Incongruity of drills with the religious and social norms of the community
		Insufficient utilization of non-governmental organizations (NGOs)	-Lack of NGO participation -Neglecting the role of students and independent experts
	**Organizing Exercise Steps**	Deficiencies in hardware and software resources for exercises	-Evaluation and Tools Lack of reliable and valid tools -Weaknesses in evaluation techniques -Insufficient training resources -Weak scenario design
		Weaknesses in the implementation frameworks of exercise standards	-Lack of reliable and valid tools -Weaknesses in evaluation techniques
		Deficiencies in exercise evaluation and debriefing processes	-Failure to develop a corrective action plan -Lack of drill evaluation and analysis

### Main results

**Fig. 2:**
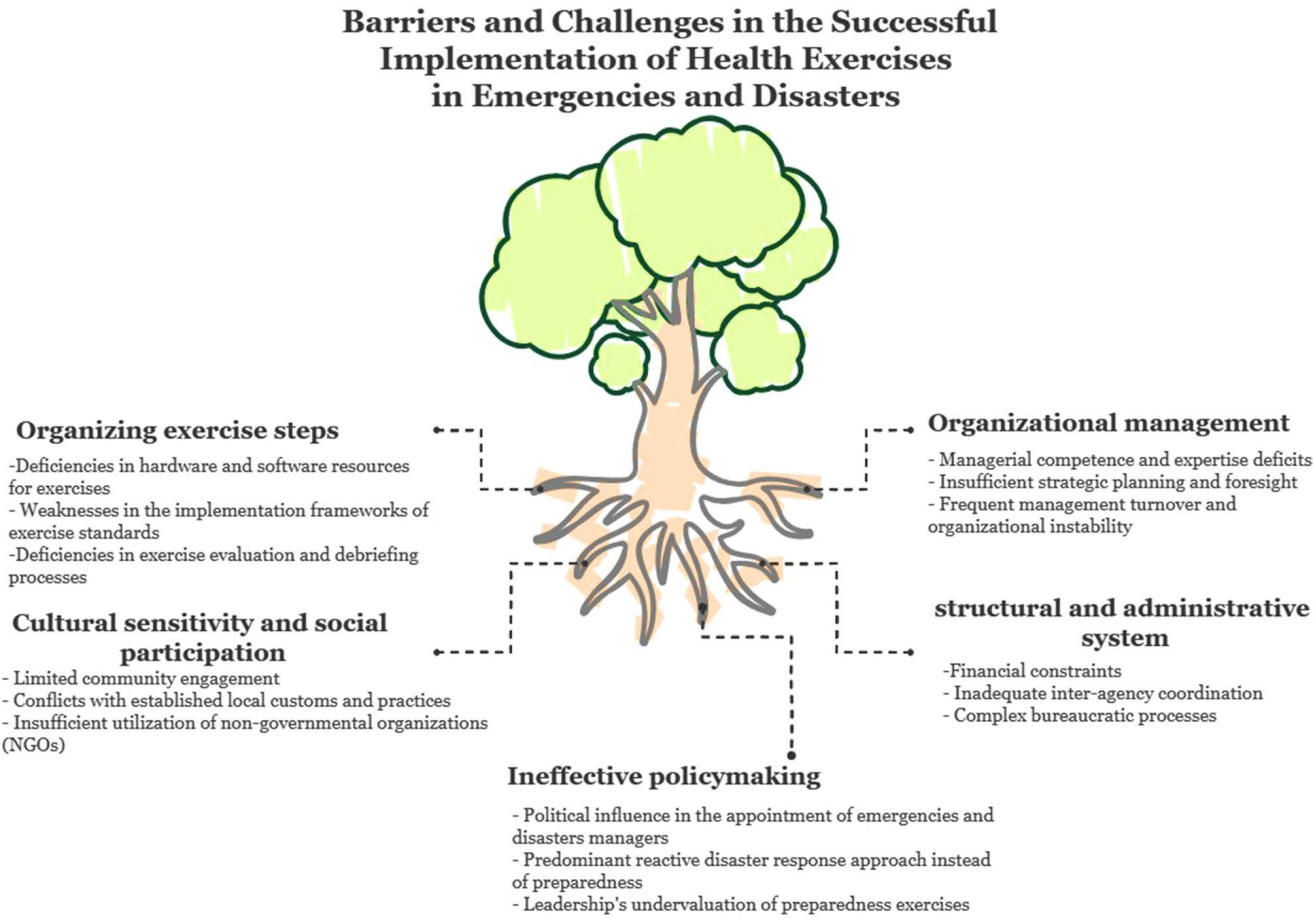
Major challenges and barriers for successful implementation of health exercises in emergencies and disasters

After multiple rounds of analyzing and summarizing the data, taking into consideration similarities and differences, one them, five main categories and 15 subcategories were created based on the results of the data analysis. The main categories included “Organizational management”, “Structural and administrative system”, “Ineffective policymaking”, “Cultural sensitivity and social participation”, and “Organizing exercise steps”. The details of the main categories and subcategories are presented in Fig. 2, Table 2.

## Organizational management

**Fig. 3:**
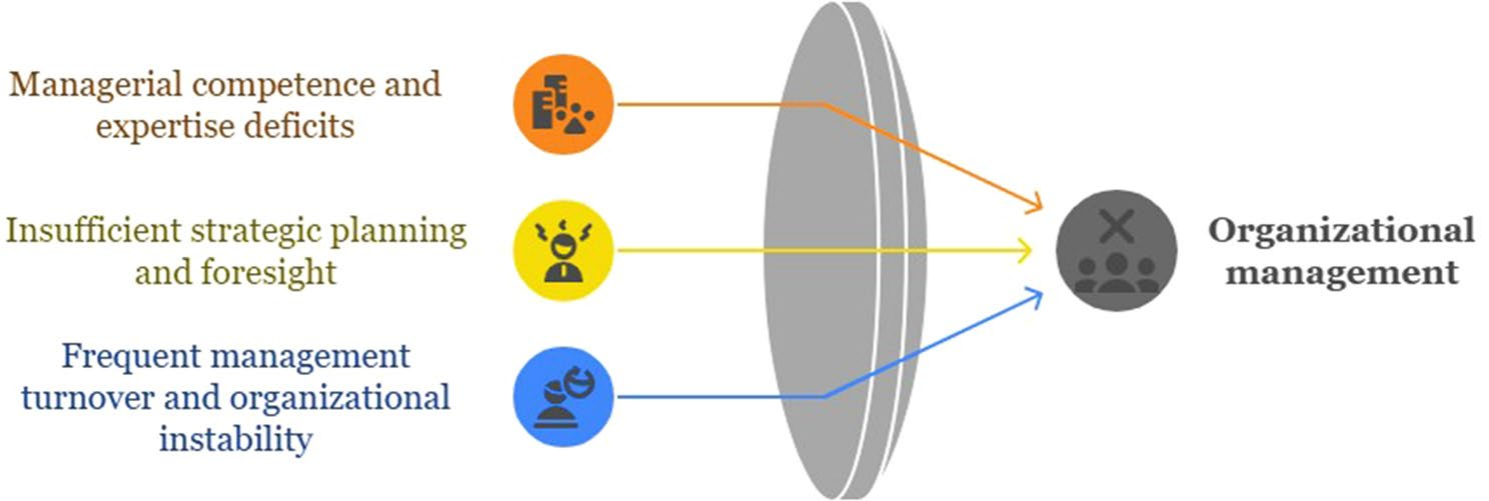
Organizational management category and relevant subcategories

Organizational management has a significant influence on the effectiveness and resilience of healthcare systems in responding to disasters. Just as organizational managers must possess the ability to identify unforeseen situations during disaster response, it is also essential for them to have an effective disaster response plan and anticipate probable scenarios for an efficient response to incidents and disasters during exercises. This encompasses the ability to shift mindsets and modify routine processes from an organizational and individual viewpoint. However, the healthcare system encounters significant challenges in planning and implementing exercises across three dimensions. The relevant subcategories of this category are presented in Fig. 3.

## Managerial competence and expertise deficits

Managers’ lack of competence and expertise is a fundamental challenge in successfully implementing disaster health exercises. The appointment of non-specialized individuals to lead disaster risk management programs, the absence of creative ideas and thinking among disaster managers, the mismatch between academic qualifications and disaster management expertise with relevant organizational positions, and the failure to employ experienced and specialized individuals for disaster management organizational roles can all pose challenges to the execution of health exercises. According to participants, managers who have not completed emergency management training courses and lack experience in participating in exercises often do not recognize the effectiveness of exercises during a response and are therefore unwilling to conduct them.

One of the interviewees says:*“Frequently*,* disaster managers are appointed to lead emergency management programs without consideration for their expertise. Often*,* they lack experience in real-world situations or even in health exercises and are unaware of the importance of exercise in enhancing the resilience of the healthcare system in responding to incidents and disasters. Consequently*,* they lack the necessary ideas for planning and implementing exercises“(p2).*

Another interviewee stated, *“Since managers do not experience specialized training on the importance of disaster exercises for achieving preparedness*,* they lack the necessary motivation and seriousness for implementing disaster exercises“ (P10).*

### Insufficient strategic planning and foresight

Exercises in the health sector are more formalistic and unable to improve the health system’s preparedness because they lack planning and foresight during disasters. Managers and employees become less motivated to conduct successful drills when drills are neglected and incentive mechanisms for conducting drills are not implemented. On the other hand, a response-oriented approach to disasters results in a lack of investment in prevention and preparedness programs, including continuous drills. Repetitive drills and a failure to address response process flaws can occasionally result from ignoring the outcomes of previous drills when planning for future organizational operations.

One of the managers of the pre-hospital emergency center says:*“Every year*,* we try to plan training programs suitable for common hazards*,* but when we approach the managers*,* they are not inclined to conduct the training and prefer to follow up on disaster response programs“(P8).*

One of the managersof the deputy of health says: *“Sometimes good exercises are held in the health sector*,* and the system’s weaknesses are identified*,* but the following year*,* the same exercise is conducted again without considering the results of past exercises and without incorporating them into the design of objectives. This leads to resource wastage and staff’s lack of enthusiasm to participate in these exercises” (P6).*

### Frequent management turnover and organizational instability

Management changes are considered one of the serious obstacles to the effective implementation of health sector exercises in disasters. Managerial stability within an organization leads to the maintenance of the organization’s readiness to respond to disasters. Frequent dismissals of managers result in disruptions in long-term planning, and often, the transfer of implicit experiences and knowledge to new managers does not occur, leading to the repetition of mistakes and wastage of resources. Disregarding specialized and experienced managers in disaster practice and management, failing to transfer knowledge and experience to new staff and managers, undermines the organization’s knowledge transfer process. Instability in management leads to interruptions in the execution of exercises and consequently weakens the health system’s preparedness against disasters.

One of the interviewees said: *“After a few years*,* every appointed manager reaches a suitable skill level in designing and executing exercises. Suddenly*,* a new manager from outside the organization is appointed. This method of dismissing managers leads to wasted resources and overlooks the time the system has been waiting to acquire specialized personnel. On the other hand*,* this sudden dismissal prevents the previous manager from sharing their documentation and experiences of executing exercises with the next person*,* which results in the loss of stability and continuity in training programs“(P20).*

## Structural and administrative system

**Fig. 4:**
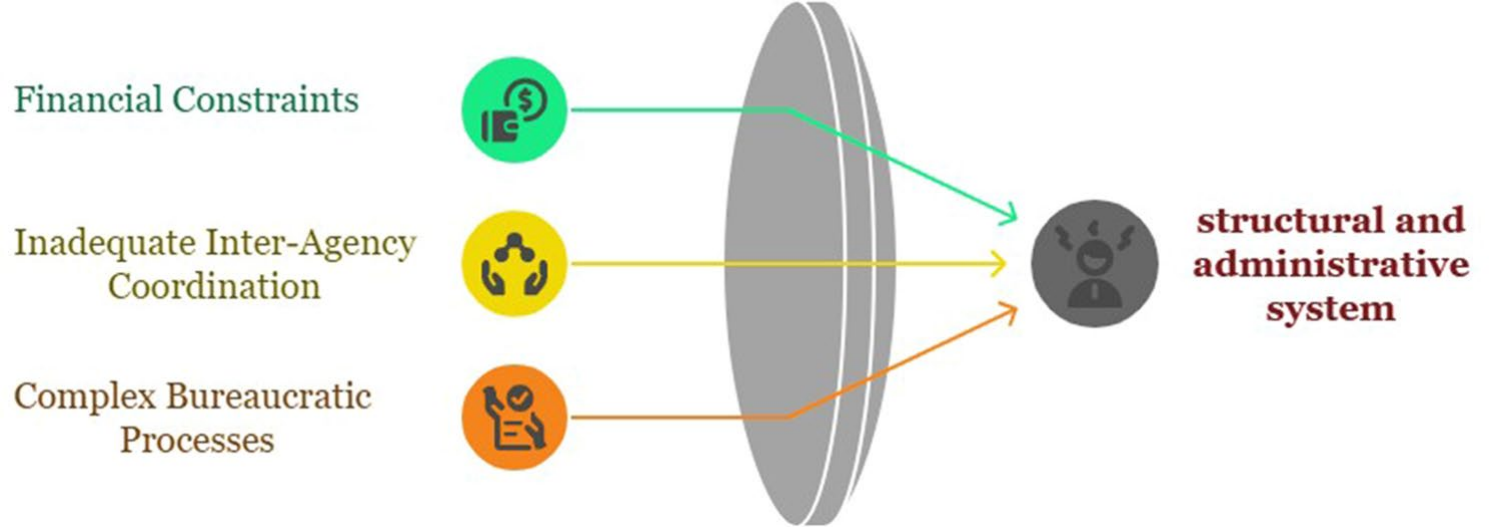
Structural and administrative system category and relevant subcategories

One of the significant challenges identified in this study regarding the conduct of the exercise was the structural and administrative system. Organizational, financial, and bureaucratic factors directly and indirectly reduce the effectiveness of the exercises by causing interruptions in their continuity. The relevant subcategories of this category are presented in Fig. 4.

### Financial constraints

The lack of financial resources in the system can hinder the implementation of simulation exercises, with execution limitations. Systems facing resource deficiencies do not prioritise prevention and preparedness programs, including exercises, in allocating funds, resulting in reduced staff training programs. Moreover, in most cases, financial resources for conducting exercises are not anticipated in the organization’s financial plans, which creates complex administrative challenges for those responsible for performing the exercises to obtain the necessary permissions for their execution.

Another interviewee says: *“When we want to conduct the exercise*,* I ask the superior for permission because the organization’s financial limits do not provide any financial resources for doing so. I am unable to hold the exercise since there is not enough money set aside for it. Practical exercises need drills and at least basic equipment*,* which requires costly resources*,* and they cannot be done around a round table“(P19).*

One of the interviewees said, *“Financial resources for conducting the exercise are sometimes allocated to the organization. However*,* the system has such a shortage of resources that the officials do not allow the allocated financial credit for the exercise to be used*,* and the predicted financial credit is spent on the system’s debts“(P16).*

### Inadequate inter-agency coordination

This sub-category refers to structural and operational challenges and inconsistencies among the organizations involved in the design and execution of emergency management exercises. The inefficiency of inter-organizational and intra-organizational communication processes leads to weaknesses in the coordination and decision-making cycle and the exchange of communications with other organizations, which stem from the lack of a clear definition of roles and responsibilities, poor coordination of human and material resources, and a lack of a shared understanding of the exercise objectives. Having a performative and symbolic view of exercise execution reduces inter-organizational cooperation, and this reduction in cooperation hinders the identification of the capacities and needs of partner organizations, causing inconsistencies in the exercise’s execution.

One of the EOC managers said:*“According to the scenario designed for the exercise*,* each organization is supposed to procure a series of equipment and supplies. However*,* this does not happen for various reasons*,* such as a lack of equipment and financial resources. If good coordination and communication had been established with partner organizations and their capacities had been identified*,* the equipment could have been procured using their resources*,* and the execution of the exercise would not have faced any issues“(P12).*

Another interviewee mentioned: *“In performing the exercise*,* like in real disasters and emergencies*,* each organization tends to take command of the incident and be at the forefront. This leads to similar work during the exercise*,* resulting in the exercise being uncoordinated with other organizations. Sometimes*,* higher supervisory organizations*,* such as the governor’s office and provincial administration*,* become involved and expect the specialized activities of the executing organization to be detailed and provided to them. Their interference reduces the quality of the exercise“(P4).*

### Complex bureaucratic processes

An inefficient administrative hierarchy creates serious obstacles for the implementation of drills. The complex and time-consuming bureaucracy for obtaining permits and financial credits leads to decreased motivation and confusion among individuals conducting the drills, which can even result in the cancellation of the drills. The lack of a unified guideline to facilitate administrative tasks and the weakness in intra-organizational coordination pose challenges to the system’s implementation of the drills.

One of the managers said:*“The process of obtaining exercise funding is very time-consuming and bureaucratic. We must submit a formal request and then get approvals from several units. Only after that does the allocation process begin*,* which sometimes does not reach us by the time of the exercise. In a few cases*,* we had to cancel the exercise altogether because funding was not secured. More time was spent on getting signatures and administrative approvals than designing the scenario. This amount of paperwork demotivates the teams. Instead of an educational and training process*,* the exercise becomes a tedious administrative project that few are ready to pursue“(P9).*

Another interviewee said, *“One of the fundamental problems is the lack of a fixed procedure for planning and executing exercises. Each organization or department has its own specific rules and regulations*,* which force us to review everything multiple times for a joint exercise. These coordinations are time-consuming and drain a lot of energy from the team*,* whereas simplifying administrative steps could make the process much easier“(P17).*

## Ineffective policymaking

**Fig. 5:**
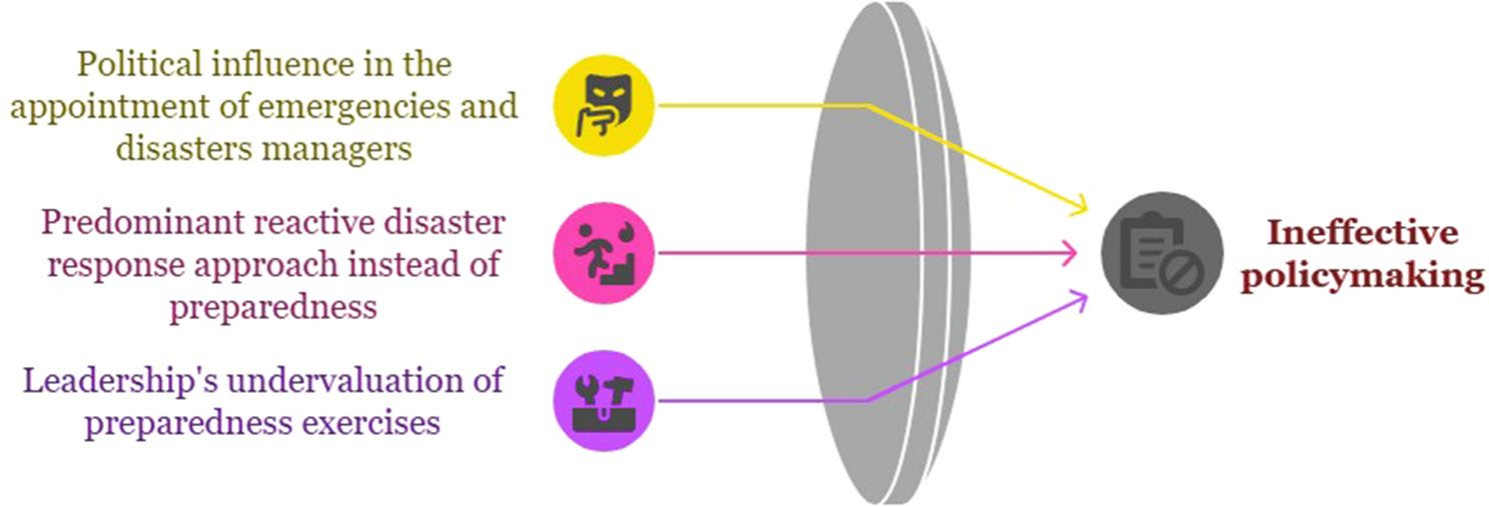
Ineffective policymaking category and relevant subcategories

The inefficacy of policies in emergency management leads to a decrease in the effectiveness of exercises and consequently weakens preparedness for responding to incidents and disasters. Political interference in appointing emergency managers and assigning specialized responsibilities to individuals lacking professional qualifications and necessary experience undermines disaster preparedness programs. On the other hand, the prevailing policy in emergency management programs focuses more on post-emergency accountability. Managers often perceive exercises as a performative and unnecessary action, overlooking the main objectives, such as assessing readiness and identifying performance gaps, which can turn drills into ceremonial and unrealistic activities. The relevant subcategories of this category are presented in Fig. 5.

### Political influence in the appointment of emergency and disaster managers

The political appointment of individuals to disaster management positions without considering their experience and expertise is one of the challenges facing the emergency management system, which severely affects the effectiveness of exercises. Political appointments negatively impact meritocracy, weaken preparedness and response programs in the long term due to management instability and bias in resource allocation, reduce stakeholder trust, and ultimately become an obstacle to the sustainable development of the health emergency management system.

One of the managers of the hospital says, *“Unfortunately*,* the selection of managers is based on their political affiliations*,* and their experience and knowledge are not considered. The result of these selections is a decrease in the quality of preparedness programs*,* including exercises. These individuals lack the necessary knowledge and skills to conduct drills. When employees see someone without a background or expertise*,* chosen based on political connections*,* taking charge of emergency management*,* they lose their motivation to participate in preparedness programs“(P11).*

Another interviewee said, *“Every time the government changes*,* all managers*,* including emergency managers*,* undergo replacement. If the selected manager has political support*,* they receive more financial and logistical resources and are more likely to allocate budgets for prevention and training programs. Constant management changes lead to disorganization and the incompletion of long-term preparedness programs*,* necessitating a restart each time“(P1).*

### Predominant reactive disaster response approach instead of preparedness

The financial resources allocated and investments in actions for the emergency response phase differ significantly from the preparedness programs. Although the economic costs associated with preparedness actions are substantially lower than those for response, since the performance of crisis managers is evaluated based on crisis response-related indicators, managers tend to allocate more resources during the response phase. Neglecting preparedness actions, including conducting drills, not only wastes resources but also increases the vulnerability of the health system.

One of the managers of the pre-hospital emergency center says, *“They have a short-term view of emergency management and only try to work on projects that yield quick results. Meanwhile*,* disaster preparedness is a long-term process“(P13).*

Another interviewee said, *“Just ask the managers about disaster preparedness. They will immediately respond that we have sufficient rescue equipment and are ready to respond. They do not understand the difference between preparedness and response*,* and their focus is on response. Showing images of rescue operations after an emergency is much more appealing than investing in preparedness drills that receive no media coverage“(P22).*

### Leadership’s undervaluation of preparedness exercises

Underestimating exercises and viewing their execution as a mere performance leads to a deviation from the main objectives of the drills, such as preparedness, inter-agency coordination, assessment of weaknesses, and enhancement of capabilities in facing emergency or challenging situations. In this case, executing exercises, intended as a learning tool, becomes a performative act and a means to satisfy officials. The misguided perspective of policymakers on conducting health sector exercises leads to a decline in the quality of the exercises, a loss of learning opportunities, and ultimately, an increased vulnerability of the health system to emergencies.

*The managers do not take the exercises seriously or believe in their usefulness. They do not adequately understand how to implement the exercises*,* perhaps due to unfamiliarity with their nature and function. The managers do not participate in the exercises and show their unimportance to the staff through their choices and views (P7).*

Another interviewee said, *“The workload assigned to managers is very high*,* leaving no time for planning and participating in exercises. Completing exercises is more of a mandatory program than a fundamental learning tool. Our presence in the exercises is only to have our names on the participants’ list; otherwise*,* we do not learn anything new“(P5).*

## Cultural sensitivity and social participation

**Fig. 6:**
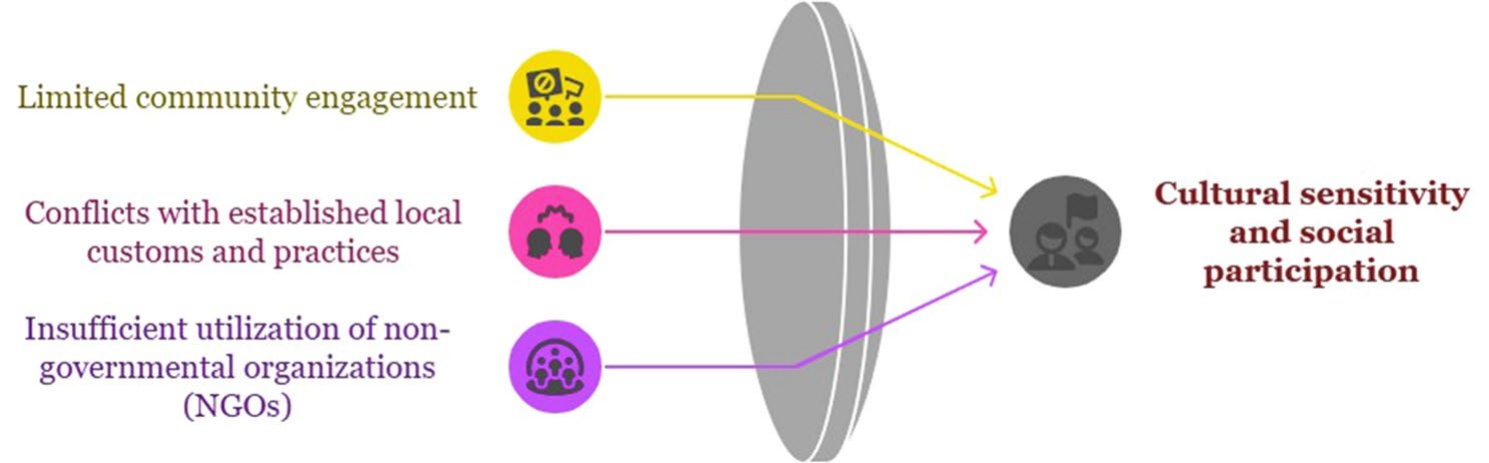
Cultural sensitivity and social participation category and relevant subcategories

Neglecting social and cultural capacities is a weakness in the design and implementation of exercises. Paying attention to social and cultural beliefs and structures, and involving communities and non-governmental organizations in exercises is one of the requirements for achieving optimal preparedness to respond to emergencies. Students, non-governmental organizations, and health professionals who operate independently play a significant role in the effectiveness of exercises. In contrast, most health systems often overlook them or do not have a defined role in the exercise process. Considering that the first responders to incidents and disasters are usually individuals and volunteer groups, empowering these individuals and involving them in health drills is essential, as it leads to improved preparedness. The relevant subcategories of this category are presented in Fig. 6.

### Limited community engagement

Neglecting the impact and role of people in the design and execution of exercises leads to disregarding local experiences, beliefs, knowledge, and local capacities for effective response to incidents and disasters. Communities do not play a critical and participatory role in the implementation of exercises, and their presence is limited to being on-site or following the orders of the drill’s executors. However, to enhance community resilience, active community participation in exercises is necessary. Ignoring and disregarding people can destroy their sense of value and instill a feeling of insignificance in their opinions during decision-making. Continuing this approach leads to public distrust of response organizations during incidents and disasters.

One of the managers of the Disaster Risk Reduction Office says,“*In most exercises*,* people are present as spectators. We asked people to attend a local drill*,* but it was unclear why they were being asked or whether they could be of help. We should seek people’s opinions and involve them in the design and execution of the exercise. People have gained experience and knowledge during previous crises*,* which is very helpful for designing an effective drill; yet*,* it is often overlooked. They may have firsthand experience with earthquakes*,* floods*,* or other emergencies*,* and we can include them in the scenarios or execution“(P3).*

Another interviewee said, *“Officials at higher levels carry out the exercise design*,* and until the implementation stage*,* the people are unaware of it. In the exercise design*,* there has always been a lack of local representatives and volunteer groups. We design exercises for communities without conducting any needs assessments with the people*,* and since there is no familiarity with the local context*,* the cultural and religious aspects are often overlooked in the exercise design. When we reach the implementation stage*,* conducting an exercise activity coincides with their religious rituals*,* which reduces public participation” (P15).*

### Conflicts with established local customs and practices

Neglecting the local cultural and social characteristics renders the exercises meaningless for the people. Paying attention to local communication patterns and traditional structures that involve the participation of elders and community leaders ensures that the exercises are conducted within the real context of the community and are welcomed by the local populations. Collaboration and participation in local communities foster a sense of trust in the organizations conducting the exercises, thereby enhancing the effectiveness of the training. Aligning the exercises with the community’s culture, while improving the quality of the provided training, strengthens the community’s connection with the organizations responding to the emergency.

One of the interviewees says:*““When we enter local and rural communities*,* meeting with the area’s seniors and informing them about the work we want to do is essential. We should ask them to accompany us in engaging the people for the exercises. We need to bridge the gap between scientific content and cultural beliefs to accept the exercises” (P18).*

### Insufficient utilization of non-governmental organizations (NGOs)

Non-governmental organizations (NGOs) are among the key capacities for responding to emergencies and disasters, and they are among the first groups to arrive at the disaster site. However, they have been excluded from the decision-making cycle during the training and preparedness phase, which is one reason for the poor execution of drills. Neglecting the importance of the role and impact of these groups leads to the weakening of the community-centered approach in disaster management. Organizations prioritize formal structures when designing and implementing exercises, often neglecting the capacities of local communities. However, these groups, with their regional knowledge, can significantly assist in localizing the exercises. Ignoring these groups means eliminating a significant part of the national capacity to face disasters. By utilizing the voluntary and specialized capacities of these non-governmental organizations, we can witness greater cohesion and more genuine community participation in the implementation of preparedness and training programs.

One of the managersof the Disaster Risk Reduction Office says:*“We design scenarios more for offices than for communities! On the other hand*,* because we think coordinating with NGOs might be difficult*,* we try to connect with accessible government organizations to plan*,* design*,* and implement the exercise sooner. We do not consider civil society and NGOs at all” (P14).*

*Another interviewee stated*,* “During the exercises I attended*,* I did not see a designated area for non-governmental organizations*,* despite these groups often engaging in field activities during a crisis. One of the weaknesses of the exercises being conducted is the focus on government organizations and the neglect of the capacity of non-governmental organizations” (P5).*

## Organizing exercise steps

**Fig. 7:**
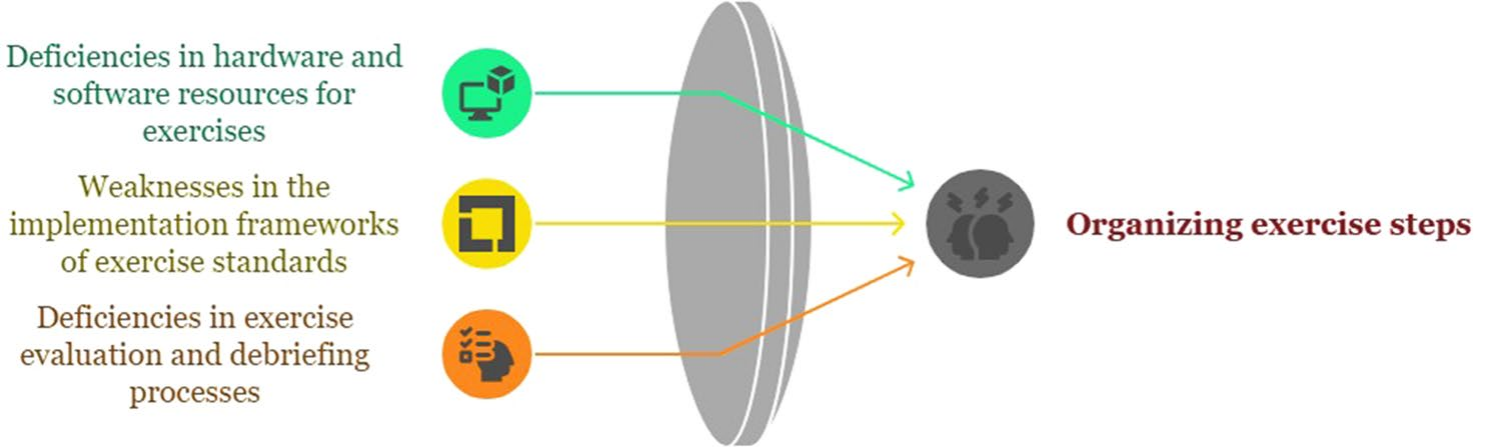
Organizing exercise steps category and relevant subcategories

Conducting preparedness exercises helps identify strengths and weaknesses within the system. Since the lessons learned and experiences gained from these exercises are used to improve operational and educational programs, merely conducting them is insufficient to enhance operational and managerial processes. Exercise designers need to be precise, and exercise organizers should plan to evaluate and follow up on the implementation of improvement programs. When managing a training program, it is essential to consider hardware and software requirements, realistic scenarios, and regular training sessions tailored to the individuals involved. Neglecting scientific documentation at the end of the exercise, skipping evaluation and hot-wash sessions, overlooking feedback, and failing to develop corrective plans indicate that the exercise has not been properly integrated into the crisis management process. The relevant subcategories of this category are shown in Fig. 7.

### Deficiencies in hardware and software resources for exercises

The lack of hardware and software equipment, including standard communication systems, medical equipment, transportation means, or the absence of virtual systems for inter-organizational communication, poses serious challenges to performing the exercise. Providing communication tools across various platforms and at all levels of the exercise, as well as utilizing social networks to establish communication between different participating organizations, is a necessity for a standard exercise that enhances coordination for incident response among the teams present.

One of the managersof the Logistics sector says, *“Forecasting and preparing logistics and supplies for the exercise are essential requirements. Transportation*,* provision of necessary items*,* and meeting basic needs such as water*,* food*,* and shelter for participants in the exercise*,* as well as creating a communication platform between various internal and external units and ensuring the safety and security of participants*,* just like in real incidents*,* are prerequisites for conducting a standard exercise” (P21).*

*Another interviewee stated*,* “We should not forget the potential of new communication platforms*,* including social networks*,* for establishing communication. With the help of these networks*,* it is possible to transfer information from the incident site quickly*,* send early warning alerts for prevention*,* and even send real-time videos from the rapid assessment team to the Incident Management System (IMS)” (P16).*

### Weaknesses in the implementation frameworks of exercise standards

The inability and weakness in applying the principles and guidelines outlined in the organization’s operational response plans, including the lack of an incident command framework, the lack of proper zoning for the exercise area to deploy units, the failure to adhere to principles and regulations by all participating organizations, and neglecting the security and safety of exercise participants, lead to a decrease in the effectiveness of the exercise. The unfamiliarity of staff with the incident command structure, neglect of individual and functional skills of participants, and the absence of clear instructions for the exercise are significant reasons for the weakness in implementing exercise structures and standards. Such exercises will not be able to enhance the system’s readiness.

One of the interviewees says:*““One of the problems we always face is the lack of a unified commander during the exercise. Each organization thinks it is responsible for commanding the stage*,* leading to overlaps in command. Instead of increasing inter-organizational coordination*,* the exercise becomes a platform for showcasing organizational conflicts. In addition to familiarizing all exercise staff*,* the personnel from other participating organizations must also be acquainted with the ICS chart” (P23).*

*Another interviewee stated*,* “During the execution of the exercise*,* we must be familiar with the zoning of the training area. We need to know where the gathering place and advanced medical posts are established. We should also be aware of the locations and activities of the participating organizations and be familiar with the zoning guidelines” (P11).*

### Deficiencies in exercise evaluation and debriefing processes

Weaknesses in the design and implementation of standard tools, the lack of evaluators familiar with evaluation principles and the health response framework, and the absence of valid indicators hinder the reliability of exercise outputs. Additionally, ignoring feedback and lacking a corrective plan, especially in the absence of a responsible person to follow up on results, leads to missed learning opportunities. The lack of scientific and documented reports from exercises disrupts the knowledge transfer process and hinders the organization from continuous progress and improvement.

One of the interviewees says:*““In various exercises*,* I have repeatedly seen that no specific tools are reliable and generalizable for evaluating the exercise. Sometimes*,* individuals unfamiliar with the response program are chosen to evaluate the exercise*,* resulting in impractical feedback” (P16).*

*Another interviewee stated*,* “We conduct exercises and evaluations. However*,* the evaluation results are not used as a basis for decision-making for future exercises. Important feedback and identified weaknesses are often overlooked*,* and even in the best-case scenario*,* where the exercise results are documented*,* they are archived. No individual or group seeks to improve the process or address the weaknesses” (P12).*

## Discussion

The present study examined the challenges of exercise management within the healthcare system. The identified challenges included “Organizational management”, “structural and administrative system”, “Ineffective policymaking”, “Cultural sensitivity and social participation”, and “organizing exercise steps”.

## Organizational management

One of the most significant identified challenges was organizational management. Most participants believed that the methods of organizational management and foresight in planning, as well as merit-based management, have a considerable impact on the effectiveness of health sector practices. Risk management involves assessing, preventing, controlling, and monitoring risks to minimize and mitigate new risks [33]. This highlights the crucial role of the leader in the healthcare system [34]. The specific competencies of managers lead to strengthening organizational capacities at all stages of risk management [35]. Lauren Walsh et. all believe that individual and family preparedness through the development of response plans, provision of equipment, and scenario drills; effective role-playing at the organizational and societal levels; maintaining situational awareness of health risks at various stages of a crisis; and establishing effective communication with credible sources while considering cultural considerations are among the key competencies in the framework of training and preparedness in the health sector for disasters, which enhance the capacity of health forces to respond to disasters. These competencies provide the necessary foundation for training and practice in disaster health management [36]. Therefore, the existence of global standards for providing uniform training to health professionals and managers is essential [37, 38]. A lack of planning and organization to address incidents, combined with insufficient training for staff to respond to emergencies and disasters, can cause irreparable damage to the country’s healthcare system. Natasha Sanchez Cristal, in her study, considered the integration of simulation-based exercises into health emergency management training programs to ensure an appropriate response during disasters as a significant essential need in planning [39]. Klassen et al. Their study also emphasized the necessity of a regular training program to prepare individuals involved in emergencies to become familiar with their actual roles and responsibilities before real disasters and to identify weaknesses in the developed programs [38]. The lack of expertise, proper training, and the development of non-technical skills for health managers and disaster responders can lead to inefficiency and jeopardize the quality of disaster preparedness and response [40]. ​ Health managers in disaster situations must possess non-technical skills, such as effective communication, situational awareness, knowledge of human resources, organizational and coordination skills, decision-making, critical thinking, and problem-solving abilities [41]. Managerial instability in the health system, combined with the lack of a straightforward succession process, imposes significant financial costs on the system and negatively impacts the morale of colleagues [42]. The authors state that in the present study, deficiencies in organizational management have also been recognized as one of the major challenges in achieving readiness and response in the health system. Effective and participatory governance, characterized by strong communication and coordination with key stakeholders, as well as the development of an organizational learning culture responsive to crises, is essential for achieving readiness and an appropriate response within the health system. It is recommended that a comprehensive training system be designed and implemented for health managers and personnel, including targeted training and disaster simulation courses, to enhance the necessary knowledge, skills, and coordination for effective crisis management in the health sector. Health systems should prioritize succession planning, knowledge, and experience management.

## Structural and administrative system

Another fundamental challenge that affected the continuity and efficiency of health sector exercises was the prevailing structural and administrative system. On the other hand, other issues, such as weak communication within and outside the organization and poor coordination between these units, result in overlapping work, wasted resources, increased injuries, and higher human casualties, which exacerbate the problems with the structural and administrative system [43]. Lack of financial funding leads to a shortage of necessary equipment for conducting exercises and a decrease in the number of exercises held, resulting in a reduction in the number of trained personnel, which in times of crisis also hinders an effective response to disasters and emergencies, as confirmed in the study by Masbi and colleagues [44]. Although the lack of access to financial resources limits the possibility of simulation, it can be partially overcome through careful planning, facilitating decision-making processes, creating flexible structures for disaster response, and employing innovative methods. Naibaho & Lutfha also considered the facilitation of complex and time-consuming processes caused by administrative bureaucracy in emergencies essential for ensuring an appropriate response in their study. They identified a reliance on traditional bureaucracy and an excessive focus on emergency regulations, which exacerbated the critical conditions [45]. The exercises conducted must address the challenge by simulating real-world conditions. The researchers of this study suggest that, considering the lack of resources, scenarios with a higher probability of occurrence and the execution of key exercises should be prioritized. We recommend using low-cost and alternative methods, strengthening interdepartmental collaboration, allocating financial resources for exercise execution, and reforming administrative processes to overcome this challenge.

## Ineffective policymaking

Another challenge identified by the participants as a fundamental and influential factor in the quality of practice was ineffective policymaking. Political appointments are one reason for the uneven distribution of resources in the healthcare system. In this case, the health system’s needs are overlooked, and the allocation of resources for crisis preparedness exercises is based on political interests. Peng Tao believes that the changes made to the country’s crisis management mechanism have a significant impact on government directives in response to incidents and disasters. This issue reflects the impact of the rule of law, which helps strengthen and stabilize the emergency management system [46]. The study by Esteban Ortiz-Prado et al. showed that appointing individuals without considering their expertise is a significant barrier to effective leadership and advancing the health system. This study emphasized the importance of specialized training and managerial skills, including advocacy for health managers, as well as structural and organizational reforms to enhance management in the health system [47]. Political influence plays a significant role in defining, financing, and implementing disaster resilience programs; however, political factors sometimes hinder the effective execution of resilience and preparedness policies [38]. On the one hand, communities’ neglect of prevention and preparedness costs, and their preference for receiving direct relief and tangible benefits after disasters, lead policymakers toward short-term and performative actions. This leads to a disproportionate allocation of resources [48]. Frequent managerial changes pose a threat to system stability and weaken decision-making processes. The short-term political outlook of managers causes them to overlook the needs of specific parts of society and to pursue and achieve short-term programs. Adverse consequences impact the health system, including significant resource wastage due to the lack of informed and logical decision-making and inadequate long-term planning. Long-term thinking and planning in the healthcare system are a grave necessity [49]. Jackson and McKay, in their study, demonstrated that sometimes the designed exercises fail to meet the desired quality. Sometimes, the goal of the exercise is not to identify weaknesses, but to ensure its successful execution. These exercises are more demonstration-oriented and lack an educational approach. The authors of this article believe that exercises are a vital tool for identifying weaknesses in the system’s responsiveness, and neglecting the main objectives of the exercises leads to a lack of system preparedness [50]. Researchers believe that to maintain strong disaster preparedness programs and enhance decision-making, it is crucial to establish stable management teams that are not influenced by political changes, select managers based on clear criteria, regularly evaluate crisis managers using specific measures, and shift the focus of decision-makers from merely practicing to conducting focused exercises.

## Cultural sensitivity and social participation

One of the most notable categories overlooked in the exercise was cultural sensitivity and social participation. This is because the way communities respond to and understand risks associated with incidents depends on the attention given to the prevailing socio-cultural structure of society [49]. Factors such as previous disaster experiences, participation in drills, familiarity with emergency response resources, and training that considers cultural characteristics, beliefs, and community risk perception can influence the level of preparedness and response in communities [51]. Various studies have shown that ignoring cultural and social norms in conducting drills can lead to reduced social participation, weakened preparedness, and distrust, emphasizing the importance of cooperation and public participation in strengthening risk management programs [52, 53].

The present study’s findings were consistent with those of Wilbroda et al. and Smith et al. These studies emphasized that exercises conducted with community participation lead to an increase in response capacity and resilience to disasters. However, the lack of communication with communities during the planning and execution of exercises results in the inability to utilize local knowledge and resources, as well as a lack of dynamism in the exercises [54]. Researchers emphasize the importance of being aware of cultural differences when planning and conducting exercises, involving the community in these processes, establishing ways to collaborate with non-governmental and community-based organizations, and acknowledging the contributions of volunteer and community groups to the exercises.

## Organizing exercise steps

The latest identified challenge was paying attention to the characteristics and conditions of conducting standard training. Insufficient support for facilities and equipment, financial constraints, lack of interdepartmental coordination, inadequate leadership attention, and insufficient training in designing training programs are factors affecting the effectiveness of the training [22]. Another training resource that directly impacts the process and flow of training is the presence of realistic and diverse scenarios. Scenarios that closely resemble reality can enhance the quality of training [55]. Using checklists and standard tools for evaluating training not only increases the accuracy of result analysis but also reduces cognitive and interpretive biases. Precisely and meticulously identifying the strengths and weaknesses of the participating teams’ performance is one of the advantages of post-training evaluation, which leads to increased disaster preparedness and reduced vulnerability [56]. To identify strengths and weaknesses, as well as to review information and analyze experiences, conducting an immediate debriefing and a delayed debriefing is essential. Conducting a post-exercise evaluation is a key indicator of the quality of the exercise [57]. Review sessions conducted after each exercise enhance the learning process and narrow the gap between theoretical knowledge and practical performance [57, 58]. Poorsiahbidi et al. in their study showed that establishing a supportive foundation for the training program, scenario design, presentations/orientation sessions, using expert evaluators, and defining an appropriate corrective action plan have the greatest impact on the success of an emergency exercise [17]. According to researchers, the health system needs to predict emergency resources and reserves to improve the quantity and quality of exercises and to respond appropriately to disasters. By conducting systematic evaluations after exercises, they can enhance the learning cycle and strengthen resilient health infrastructure. Providing adequate facilities and equipment for emergency exercises, implementing educational programs, conducting continuous and standardized exercises, and ensuring that human resources are empowered to take on various roles in drills, such as evaluator and controller, are crucial for increasing the efficiency and effectiveness of the exercises.

## Limitations and strengths of the research

The strength of this study is the diversity of participants from different sections of the health system, including prehospital, hospital, eoc, disaster risk reduction office, health sectors and deputies of logistic in the different universities of medical sciences in Iran.

This study has several limitations that should be acknowledged. First, the study’s limitation it was that all participants’ opinions and statements were assumed to be honest but the mental and physical conditions of the individuals may have influenced the way they responded and if individuals reported unrealistic responses due to being in adverse physical and mental conditions this was beyond the researcher’s control.Second, The present study provides insights into the rich experiences of health system managers in Iran. However, it is important to note that the results may not be generalizable to all cultures and contexts. The experience of designing, conducting and evaluation of disaster exercises (discussion-based and operations-based) in emergencies and disasters can be influenced by cultural, economic, social, and educational factors. Therefore, it is recommended to conduct similar studies in other communities to gain a more comprehensive understanding of health system managers experiences in field of management of exercises in emergencies and disasters.

## Practice and policy implications

This research presents a comprehensive framework for addressing the challenges of conducting exercises in the health sector, which includes five principal axes: The first axis, organizational management, emphasizes succession planning and knowledge management. We propose the development of a comprehensive educational system specifically for health exercises as an innovative solution to address the challenges associated with organizational management. The second axis seriously reviews the administrative system, sustainable financing, and inter-organizational strengthening. With a long-term perspective, the third axis focuses on the predominance of long-term planning and systems thinking over short-term and periodic actions. The fourth axis, with an approach to localizing exercises, emphasizes the importance of understanding cultural and religious differences and involving local and spiritual communities to enhance the effectiveness of the exercises. The fifth axis states that incorporating disaster management exercises into the regular process requires a thorough approach based on scientific principles, while also identifying and addressing any gaps to continually improve and build resilience.

## Conclusion

This study identifies critical challenges and obstacles encountered by the health system in the design, implementation, and evaluation of health sector exercises for emergencies and disasters. The management of these exercises within the Iranian health system is subject to numerous difficulties. Key challenges include ineffective and inappropriate policymaking, insufficient consideration of cultural sensitivity and social participation, and complex structural and administrative bureaucratic processes. Understanding the obstacles and challenges in implementing discussion-based and operations-based exercises in emergencies and disasters derived from the experiences of exercise implementers in the health system can help health planners and managers proactively address these obstacles and by considering these challenges and striving to reduce them before designing an exercise they can maintain and improve preparedness of their community’s healthcare system through the implementation of successful and effective exercises. Future research should focus on gathering insights from experts and experienced individuals in the field of disaster exercise implementation to explore practical solutions for overcoming the obstacles and challenges that the health system faces in optimizing the management of disaster exercises.

## Data Availability

Given the qualitative nature of this study, raw data, such as interview transcripts and field notes, cannot be shared publicly to protect participant confidentiality. Summaries of the analytic process and illustrative excerpts are provided within the article. You can find additional details from the corresponding author when you’re looking for them.
